# Pelvic Floor Workout for Preventing Stress Urinary Incontinence in Primiparous Women: A Randomized Clinical Trial

**DOI:** 10.1001/jamanetworkopen.2026.7132

**Published:** 2026-04-15

**Authors:** Lei Gao, Hongmei Zhu, Xiaohui Sun, Shiyan Wang, Bing Xie, Huixin Liu, Wenhui Ren, Jinyu Liang, Xiaoke Tang, Min Zhen, Guizhu Wu, Baoling Qin, Yan Hu, Lingrui Kong, Weipeng Chen, Xiuli Sun, Jianliu Wang

**Affiliations:** 1 Department of Obstetrics and Gynecology, Peking University People’s Hospital, Beijing, China; 2 The Key Laboratory of Female Pelvic Floor Disorders, Peking University People's Hospital, Beijing, China; 3 Department of Clinical Epidemiology and Biostatistics, Peking University People’s Hospital, Beijing, China; 4 Henan Key Laboratory of Fertility Protection and Aristogenesis, Luohe Central Hospital of Henan Province, Luohe, China; 5 Department of Obstetrics and Gynecology, Luohe Central Hospital of Henan Province, Luohe, China; 6 Department of Obstetrics and Gynecology, Zhengzhou Central Hospital Affiliated to Zhengzhou University, Zhengzhou, China; 7 Department of Obstetrics and Gynecology, Fangshan District, Beijing Maternal and Child Health Hospital, Beijing, China; 8 Obstetrics and Gynecology Hospital, School of Medicine, Tongji University Shanghai First Maternity and Infant Hospital, Shanghai, China; 9 Department of Obstetrics and Gynecology, Beijing Fengtai District Maternal and Child Health Hospital, Beijing, China; 10 Obstetrics and Gynecology Center, Peking University Shenzhen Hospital, Shenzhen, China; 11 Department of Obstetrics and Gynecology, Capital Medical University Mentougou Teaching Hospital, Beijing, China; 12 Department of Obstetrics and Gynecology, Peking University International Hospital, Beijing, China

## Abstract

**Importance:**

Rehabilitation training methods for stress urinary incontinence (SUI) need to be based on a global postural exercise, involving overall core muscle strength, core stability, and balance of the pelvis and spine. The pelvic floor workout (PEFLOW) program was developed to promote in-pregnancy pelvic floor muscle training to prevent postpartum SUI.

**Objective:**

To assess the effectiveness of PEFLOW in preventing postpartum SUI.

**Design, Setting, and Participants:**

This multicenter randomized clinical trial was conducted across 9 hospitals in China. Eligibility criteria were primiparous women aged 20 to 40 years, with singleton pregnancy of less than 16 weeks’ gestation, and who visited the outpatient obstetric department of 1 of the 9 hospitals from August 1, 2020, to June 6, 2022. Participants, who were followed up through January 17, 2024, were randomly assigned to the exercise group or the control group and were followed up at 37 weeks’ gestation and 6 weeks, 3 months, 6 months, and 12 months post partum. Primiparous women with severe complications; SUI or pelvic organ prolapse; and/or a history of cervical insufficiency, recurrent miscarriage, or induced labor were excluded. Intention-to-treat (ITT) analysis was conducted between May 1 and July 1, 2024.

**Intervention:**

Participants in the exercise group were trained to practice PEFLOW from 28 weeks’ gestation to delivery, with checkups and guidance provided every 2 weeks. Participants in the control group were provided usual medical care.

**Main Outcome and Measure:**

The primary outcome was the incidence of SUI at 6 weeks post partum among participants in the exercise and control groups. SUI was diagnosed based on urinary leakage when abdominal pressure increased or detected from a stress test. Symptomatic SUI and the Modified Oxford Scale (MOS) score were recorded.

**Results:**

A total of 764 women (median [IQR] age, 29 [27-32] years) were randomized into the exercise group (n = 382) and the control group (n = 382). ITT analysis showed that the incidence of SUI at 6 weeks post partum was significantly lower in the exercise group than the control group (8.7% [32 of 367] vs 13.9% [50 of 360]), with a risk difference of 5.17 (95% CI, 0.36-10.03) percentage points (*P* = .03). The proportion of women with an MOS score of 4 or higher at 6 weeks post partum was significantly higher in the exercise group than the control group (17.8% [68 of 382] vs 7.9% [30 of 382]), with a risk difference of 9.95 (95% CI, 5.05-14.87) percentage points (*P* < .001).

**Conclusions and Relevance:**

In this multicenter randomized clinical trial, PEFLOW effectively reduced SUI incidence at 6 weeks post partum. This finding suggests that PEFLOW is effective in preventing SUI in pregnancy.

**Trial Registration:**

Chinese Clinical Trial Registry Identifier: ChiCTR2000029618

## Key Points

**Question:**

Is a pelvic floor muscle training program, called pelvic floor workout (PEFLOW), effective in preventing postpartum stress urinary incontinence (SUI)?

**Findings:**

In this randomized clinical trial including 764 primiparous women, participants in the exercise group had a significantly lower incidence of SUI than participants in the control group at 6 weeks post partum (8.7% vs 13.9%).

**Meaning:**

The finding of this study suggests that PEFLOW effectively reduced postpartum SUI incidence and may prevent SUI in pregnancy.

## Teaser

This randomized clinical trial evaluates whether a pelvic floor workout decreases the incidence of stress urinary incontinence among pregnant and postpartum women in China.

## Introduction

Stress urinary incontinence (SUI), defined as involuntary leakage of urine during increased abdominal pressure,^1^ adversely affects women’s health and quality of life. The incidence of SUI at 8 weeks post partum was reported to be 25.7%.^2^ Pregnancy and delivery play important roles in the development of SUI by weakening the pelvic hammock.^3,4,5^ However, there is no consensus on effective prevention strategies for SUI in pregnant women.

Pelvic floor muscle training (PFMT) is recommended as the first-line treatment for SUI.^6^ However, the effectiveness of prenatal PFMT remains inconclusive. Reilly et al^7^ concluded that SUI occurrences in the third month post partum were lower among women who performed PFMT regularly than among those who did not. Conversely, based on a meta-analysis of randomized clinical trials (RCTs) on PFMT, Boyle et al,^8^ suggested that PFMT during pregnancy does not prevent urinary incontinence. Inconsistent conclusions across studies might result from variations in procedural appropriateness and patient adherence.^9,10,11,12,13^ Determinants of PFMT’s effectiveness for pregnant women, such as the optimal number of pelvic floor muscle (PFM) contractions, duration of PFMT, and frequency of PFMT, need to be clarified.

In 1981, Souchard first developed global postural exercise, an exercise program designed to restore muscular strength by having women maintain normal posture over time.^14^ Global postural exercise aims to correct postural misalignments by stretching muscle chains. A previous study demonstrated that the spinopelvic skeletal shape was closely related to pelvic floor dysfunction.^15^ Fozzatti et al^14,16^ demonstrated that both global postural exercise and PFMT were effective for treating SUI, with global postural exercise being substantially more effective.

To explore a more effective exercise program for SUI prevention, we integrated PFMT into global postural exercise at various intensities and developed a training program called pelvic floor workout (PEFLOW). The postures in PEFLOW were specifically designed for women to practice during pregnancy and the postpartum period. Taking patient compliance into consideration, we developed an application that incorporates the PEFLOW procedures and procedural requirements, instructions, and a programmable reminder system, aiming to facilitate women’s regular PEFLOW practice at home or in any private place with the highest time flexibility and maximum program compliance.^17,18^ An online supervision module was also integrated into the program to record patient compliance with their consent. To assess the effectiveness of PEFLOW in preventing postpartum SUI, we designed and conducted a multicenter trial.

## Methods

### Study Design

The study was a 2-arm, parallel, multicenter RCT conducted across 9 tertiary or district hospitals focusing on maternal and infant health care in China, as described previously.^19^ The institutional review board of Peking University People’s Hospital approved the trial protocol (Supplement 1). All participants provided written informed consent. We followed the Consolidated Standards of Reporting Trials (CONSORT) reporting guideline.

### Participants

Participants were recruited from primiparous women who visited the outpatient obstetric department of 1 of the 9 participating hospitals from August 1, 2020, to June 6, 2022. These patients were eligible for inclusion if they were between 20 and 40 years of age, pregnant with a single fetus of less than 16 weeks’ gestation, capable of understanding the research procedures, and able to provide informed consent in writing. Patients were excluded if they had severe complications that the intervention may exacerbate; a history of SUI or pelvic organ prolapse; and/or a history of cervical insufficiency, recurrent miscarriage, or induced labor. Participants were followed up through January 17, 2024.

Sample size was calculated using Power Analysis and Sample Size Software 2019 (NCSS) and was based on a reported 8-week postpartum SUI occurrence of 25.7%.^2^ We hypothesized that SUI occurrence at 6 weeks post partum would be close to 25.7% and that SUI occurrence in the exercise group could decline to 15%. Anticipating a 10.7%–percentage point difference in occurrence between the exercise and control groups, we needed to enroll a total of 586 participants (293 per group) to provide 90% statistical power at a 2-sided significance level of .05. Accounting for a potential 20% dropout rate, we decided to recruit at least 734 participants (367 in each group) for the trial.

### Randomization

An independent methodological statistician, not involved in recruitment, follow-up, or data analysis, generated a simple randomization sequence using a computer random-number generator. A single common sequence was used for all centers. To ensure allocation concealment, this sequence was implemented using sequentially numbered, opaque, sealed envelopes. A dedicated research staff member, separate from the recruiters, safeguarded all envelopes. After the baseline data were collected at enrollment, participants were randomly assigned to either the exercise group or the control group at a 1:1 ratio via sealed envelopes (Figure). Since it was not feasible to blind the participants and physiotherapists involved in this trial, we trained all investigators on the study components, implementation requirements, detailed procedures, and precautions to minimize study biases. Investigators who conducted patient examinations, evaluations, and data analysis were blinded to the grouping information.

**Figure.**
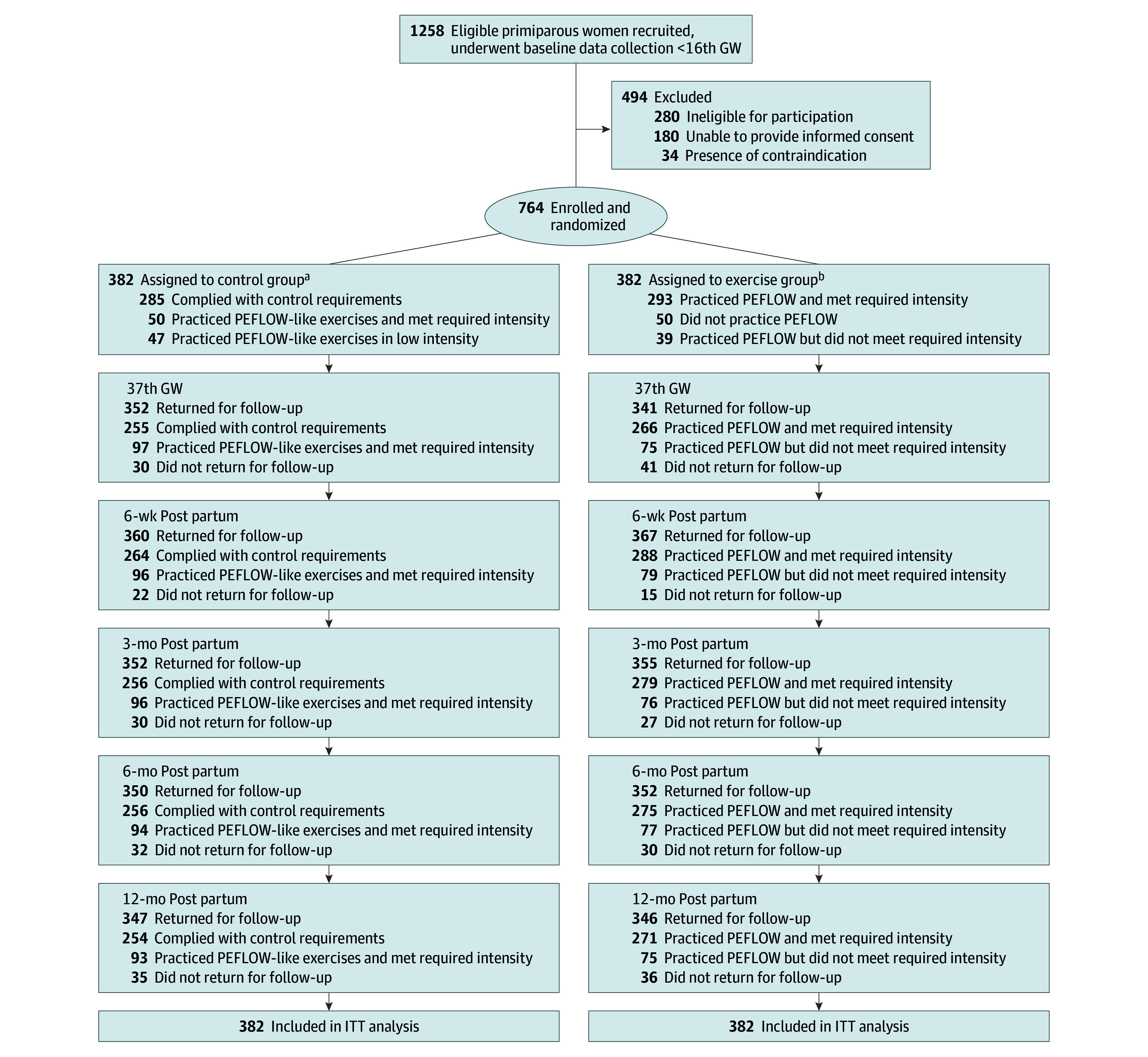
Flowchart of Screening, Randomization, and Analysis Populations ITT indicates intention to treat. ^a^Participants in the control group received obstetric examinations every 2 weeks from the 28th gestational week (GW) to delivery. Among 382 participants in the control group, 370 had delivery and neonatal outcomes. ^b^Besides regular obstetric examination, participants in the exercise group performed regular exercises following the pelvic floor workout (PEFLOW) from 28th GW to delivery. Among 382 participants in the exercise group, 375 had delivery and neonatal outcomes.

### Intervention

Baseline information was collected at enrollment from participants in both groups. Key factors included age, prepregnancy body mass index, educational level, occupational activity category, dominant working posture, history of constipation, dominant defecation posture, family history of SUI, and score on the Modified Oxford Scale (MOS, with a score of 0 indicating no; 1, flicker; 2, weak; 3, moderate; 4, good; and 5, strong contraction^20^).

Interventions for participants in the exercise group began with physiotherapists guiding them to perform PEFLOW. The exercise group engaged in PEFLOW from 28 weeks’ gestation until delivery, following a specially designed video guide and application available for download to mobile devices. The application provided health education on pelvic floor protection and the procedural video guided the PEFLOW exercises. Included in the video were 2 programs for PFMT and global postural exercise. PEFLOW consists of daily PFMT and 2 sessions of global postural exercises per week.

PFMT emphasizes the voluntary contraction and relaxation of the PFM surrounding the urethra, vagina, and rectum. It instructs exercisers to synchronize the contraction and relaxation of the PFM with their breathing, specifically through exhalation and inhalation, while avoiding engagement of the abdominal muscles. In the initial PFMT section, participants in the exercise group were required to repeat the primary procedure 5 times: contract the PFM with moderate strength and hold for 6 to 8 seconds, followed by 6 to 8 seconds of relaxation. After completing this section, participants performed 8 repetitions of the second section consisting of 5 maximum PFM contractions and relaxations as quickly as possible within 1 second, followed by 10 seconds of relaxation. The first and second PFMT sections together composed 1 complete PFMT set.

In this trial, global postural exercise consisted of 4 groups of postures with intensities progressively increasing from level 1 to level 4, specifically designed to match the specific gestational week. Each posture group includes 4 postures at the same intensity level lasting approximately 30 minutes. Deep breathing at a personal rhythm while maintaining the posture and holding the maximal contraction of the PFMs during exhalation is essential. Participants in the exercise group were encouraged to record the duration for each posture.

Participants in the exercise group were required to perform the overall PEFLOW program in 4 episodes: 28 to 30, 31 to 33, 34 to 36, and 37 to 40 weeks’ gestation. In each episode, participants were instructed to begin the PEFLOW session in postures at level 2 intensity. The intensity levels of the postures were adjusted upward or downward based on the Rating of Perceived Exertion of 15 or lower or higher than 15, which indicated whether the participant could or could not, respectively, activate the required muscle contraction while maintaining the current posture.

In the published trial protocol,^19^ we originally planned for the exercise group to practice PEFLOW sessions once at home with guidance from the video and once at a study site under the supervision of a physiotherapist each week. Given safety and health concerns during the COVID-19 pandemic, we modified the protocol to deliver postural and procedural regulations online via video conferencing (Zoom; Zoom Communications Inc) to avoid risk of COVID-19 transmission. Postural regulations were also conducted during each participant’s in-clinic pregnancy checkups every 2 weeks, during which data on the PEFLOW practice were collected for procedural evaluation. No video or audio was recorded during online instructions to protect participants’ privacy. Any participant experiencing unusual symptoms (eg, abdominal pain, vaginal bleeding, or abnormal fetal movement) would be referred to the obstetric department for assessment of whether she should continue with the intervention. In such a situation, an anticipated adverse event report would be prepared and forwarded to the principal investigator (X.S.).

The intervention for participants in the control group consisted of usual medical care. They received an obstetric examination every 2 weeks from 28 weeks’ gestation to delivery.

Data on exercise status, including whether participants exercised and the duration and weekly frequency of exercise, were collected from both the exercise and control groups during each of the 2-week pregnancy checkups. Private interviews were conducted with participants in the control group to determine whether any had engaged in PEFLOW, given the unfeasibility of group blinding. Participants in the control group were categorized into those who did not engage in PEFLOW or those who engaged in PEFLOW-like exercises and met 50% or more of the required exercise duration or intensity. Participants in the exercise group were categorized into those who practiced PEFLOW and met or did not meet 50% or more of the required exercise duration or intensity.

Outcomes were assessed for participants in the exercise and control groups at 28 weeks’ gestation and at 6 weeks, 3 months, 6 months, and 12 months post partum. During these periods, data on PFM strength measurements and SUI symptoms were collected privately as the outcome indicators.

### Outcomes

The primary outcome of this study was the incidence of SUI at 6 weeks post partum. The secondary outcomes were (1) the incidence of SUI at 37 weeks’ gestation and at 3 months, 6 months, and 12 months post partum and (2) PFM strength at 37 weeks’ gestation and at 6 weeks, 3 months, 6 months, and 12 months post partum.

SUI was diagnosed if the stress test results were positive for involuntary urinary leakage during a Valsalva maneuver or coughing in the lithotomy position^21^ and/or if the response to the question “When does urine leak?” was urinary leakage during coughing, sneezing, and/or physical exercise. This determination was in accordance with the International Consultation on Incontinence Questionnaire–Urinary Incontinence Short Form (score range: 0-21, with the highest score indicating severe urinary incontinence).^22^ PFM strength during contraction was measured by digital vaginal examination using the 5-point MOS.^20^

### Statistical Analysis

When conducting comparisons, we used frequencies and percentages to represent categorical variables. Continuous variables were summarized using means (SD) or medians (IQRs). The Kolmogorov-Smirnov test was conducted to assess the normality of continuous variables.

The primary and secondary outcomes were analyzed using intention-to-treat (ITT) methods. ITT analyses of the primary outcome were conducted in 3 dimensions: (1) multiple imputation was performed using logistic regression with 5 imputations, using the complete variables as auxiliary variables, and combining the multiply imputed datasets using the Rubin formula; (2) conservative imputation was conducted by assigning the worst outcome to cases with relevant missing data; and (3) complete case analysis was performed without imputation. ITT analyses of secondary outcomes were conducted based on conservative imputation. The differences in SUI incidence and proportion of MOS score of 4 or higher (indicating good to strong contraction) between the exercise group and the control group at different time points were analyzed using generalized estimating equations. Analysis of SUI incidence across different exercise intensity levels in the exercise group was performed using generalized estimating equations, which were based on complete data cases. The 95% CI for the difference between the 2 groups was calculated using the Wilson procedure with a continuity correction. The χ^2^ test or Fisher exact test was applied to compare the differences in proportions between the 2 groups.

Data analysis was conducted between May 1 and July 1, 2024, using SPSS 27.0 (IBM) and GraphPad Prism 9.5.0 (GraphPad Software). A 2-sided *P* < .05 was considered to be statistically significant.

## Results

From August 1, 2020, to June 6, 2022, 1258 primiparous women were recruited from 9 centers. After 494 were excluded, 764 women (median [IQR] age, 29 [27-32] years) were found to be eligible, enrolled, and then randomly assigned in a 1:1 ratio to either the exercise group or the control group. The baseline characteristics of all participants are provided in Table 1. A total of 367 participants in the exercise group and 360 participants in the control group returned for follow-up at 6 weeks post partum (Figure).

**Table 1.** Participant Baseline Characteristics

Characteristic	Participants, No. (%)	
	Exercise group (n = 382)	Control group (n = 382)
Age, median (IQR), y	30 (27-32)	29 (27-32)
Prepregnancy BMI, median (IQR)	20.90 (19.40-22.80)	21.00 (19.50-23.00)
Educational level		
≥Bachelor’s degree	356 (93.19)	356 (93.2)
High school diploma	16 (4.2)	21 (5.5)
Middle school	10 (2.6)	4 (1.0)
Unspecified	0	1 (0.3)
Occupational activity category		
Low^a^	371 (97.2)	370 (96.9)
Intermediate^b^	9 (2.4)	10 (2.6)
High^c^	2 (0.5)	1 (0.3)
Unspecified	0	1 (0.3)
Dominant working posture		
Sitting	324 (84.8)	316 (82.7)
Standing	37 (9.7)	37 (9.7)
Unspecified	21 (5.5)	29 (7.6)
Constipation history		
Yes	38 (9.9)	30 (7.9)
No	343 (89.8)	348 (91.1)
Unspecified	1 (0.3)	4 (1.0)
Dominant defecation posture		
Sitting	297 (77.7)	307 (80.4)
Squatting	78 (20.4)	64 (16.8)
Unspecified	7 (1.8)	11 (2.9)
SUI family history		
Yes	21 (5.5)	18 (4.7)
No	248 (64.9)	228 (59.7)
Unspecified	113 (29.6)	136 (35.6)
MOS score^d^		
≤3	182 (47.6)	188 (49.2)
≥4	194 (50.8)	190 (49.5)

Abbreviations: BMI, body mass index (calculated as weight in kilograms divided by height in meters squared); MOS, Modified Oxford Scale; SUI, stress urinary incontinence.

^a^ Defined as light activity of the hands or legs.

^b^ Defined as continuous movement of the hands and arms, arms and legs, and arms and trunk.

^c^ Defined as movement with loads or great intensity of excavation.

^d^ MOS scores: 0 indicating no; 1, flicker; 2, weak; 3, moderate; 4, good; and 5, strong contraction.

### Primary Outcome

Based on the complete case analysis, the ITT analysis showed that SUI incidence at 6 weeks post partum in the exercise group was lower than in the control group (8.7% [32 of 367] vs 13.9% [50 of 360]), with a between-group risk difference (RD) of 5.17 (95% CI, 0.36-10.03) percentage points (*P* = .03). The ITT analysis using multiple imputation and conservative imputation also showed the same outcome (9.0% [34 of 382] vs 14.2% [54 of 382]; RD, 5.18 [95% CI, 0.66%-9.71%] percentage points; *P* = .03) (Table 2).

**Table 2.** Primary Outcome: Effect of PEFLOW on Incidence of Stress Urinary Incontinence at 6-wk Postpartum Follow-Up^a^

Analysis	SUI incidence, No./total No. (%)		Risk difference (95% CI), percentage points^b^	*P* value^c^
	Exercise group	Control group		
Multiple imputation	34/382 (9.0)	54/382 (14.2)	5.18 (0.66-9.71)	.03
Conservative imputation	47/382 (12.3)	72/382 (18.8)	6.54 (1.21-11.86)	.01
Complete case	32/367 (8.7)	50/360 (13.9)	5.17 (0.36-10.03)	.03

Abbreviations: PEFLOW, Pelvic Floor Workout; SUI, stress urinary incontinence.

^a^ The primary outcome was analyzed using intention-to-treat method.

^b^ The 95% CI for the difference between the 2 groups was calculated using the Wilson procedure with a continuity correction.

^c^ χ^2^ Test was applied to compare the differences in proportions between the 2 groups.

### Secondary Outcomes

Based on conservative imputation, SUI incidence in the exercise group was 22.0% (84 of 382) at 37 weeks’ gestation and 11.0% (42 of 382) at 3 months, 13.1% (50 of 382) at 6 months, and 19.4% (74 of 382) at 12 months post partum. All of these incidence rates were significantly lower than those in the control group at the same time points: 25.7% (98 of 382), 17.8% (68 of 382), 18.8% (72 of 382), and 29.8% (114 of 382), respectively. The between-group RDs were 3.66 (95% CI, −2.56 to −9.86) percentage points (*P* = .23), 6.81 (95% CI, 1.64-11.97) percentage points (*P* = .007), 5.76 (95% CI, 0.37-11.13) percentage points (*P* = .03), and 10.47 (95% CI, 4.18-16.66) percentage points (*P* < .001), respectively (Table 3).

**Table 3.** Secondary Outcomes

Outcome	Participants, No./total No. (%)		Risk difference (95% CI), percentage points^a^	*P* value^b^
	Exercise group	Control group		
**SUI incidence**				
37-wk Gestation	84/382 (22.0)	98/382 (25.7)	3.66 (−2.56 to −9.86)	.23
3-mo Post partum	42/382 (11.0)	68/382 (17.8)	6.81 (1.64 to 11.97)	.007
6-mo Post partum	50/382 (13.1)	72/382 (18.8)	5.76 (0.37 to 11.13)	.03
12-mo Post partum	74/382 (19.4)	114/382 (29.8)	10.47 (4.18 to 16.66)	<.001
**MOS score ≥4**				
37-wk Gestation	194/382 (50.8)	190/382 (49.7)	1.05 (−6.20 to 8.28)	.78
6-wk Post partum	68/382 (17.8)	30/382 (7.9)	9.95 (5.05 to 14.87)	<.001
3-mo Post partum	98/382 (25.7)	85/382 (22.3)	3.4 (−2.84 to 9.61)	.27
6-mo Post partum	126/382 (33.0)	104/382 (27.2)	5.76 (−0.93 to 12.38)	.08
12-mo Post partum	126/382 (33.0)	105/382 (27.5)	5.5 (−1.20 to 12.13)	.10

Abbreviations: MOS, Modified Oxford Scale; SUI, stress urinary incontinence.

^a^ The 95% CI for the difference between the 2 groups was calculated using the Wilson procedure with a continuity correction.

^b^ Analyses of secondary outcomes were based on conservative imputation. It was conducted with the worst outcome assigned to cases with the relevant missing data. Analyses of SUI incidence and proportions of MOS score of 4 or higher were performed using generalized estimating equations with repeated measures. χ^2^ test was applied to compare the differences in proportions between the 2 groups.

We also collected data on the MOS score from participants in the exercise and control groups. As shown in Table 3, the proportion of participants with an MOS score of 4 or higher (indicating good to strong contraction) at 6 weeks post partum was significantly higher in the exercise group than the control group (17.8% [68 of 382] vs 7.9% [30 of 382]), with an RD of 9.95 (95% CI, 5.05-14.87) percentage points (*P* < .001).

### PEFLOW Adherence and SUI Incidence

When regrouping the participants in the exercise group by different exercise intensities (eFigure in Supplement 2), there were several findings. First, the SUI incidence among participants with an exercise intensity level of 80% or greater was significantly lower than among those with an exercise intensity level between 50% and less than 80% at 37 weeks’ gestation (7.8% vs 16.8%), 6 months post partum (2.7% vs 11.9%), and 12 months post partum (6.9% vs 16.9%), with significant RDs of 9.00 (95% CI, 0.56-17.76) percentage points (*P* = .02), 9.22 (95% CI, 2.61-16.72) percentage points (*P* = .003), and 9.95 (95% CI, 1.76-18.59) percentage points (*P* = .01), respectively. Second, the SUI incidence among participants with an exercise intensity level of 80% or greater was lower than among those with an exercise intensity level between 50% and less than 80% at 6 weeks (8.6% vs 11.0%) and 3 months post partum (2.7% vs 7.6%), but with no significant differences. Third, the SUI incidence in both intensity groups rebounded at 12^-^months post partum.

### Maternal-Fetal Safety

Maternal-fetal health-related data are shown in Table 4. Premature rupture of membranes was reported in both exercise and control groups (17.6% [66 of 375] and 23.5% [87 of 370]). Fetal distress was observed only in the control group. Only 1 woman (0.3%) reported postpartum hemorrhage in the exercise group compared with 14 women (3.8%) in the control group. No PEFLOW-related adverse event was observed.

**Table 4.** Maternal and Fetal Safety Assessment^a^

Safety indicators	Participants, No./total No. (%)	
	Exercise group	Control group
Premature rupture of membranes	66/375 (17.6)	87/370 (23.5)
Hypertensive disorders of pregnancy	22/375 (5.9)	15/370 (4.1)
Fetal distress	0/375 (0.0)	3/370 (0.8)
Postpartum hemorrhage	1/375 (0.3)	14/370 (3.8)
Vaginal delivery	224/375 (59.7)	222/370 (60.0)
Episiotomy	91/224 (40.6)	98/222 (44.1)
Vaginal laceration	146/224 (65.2)	151/222 (68.0)
Forceps delivery	15/224 (6.7)	16/222 (7.2)
Fetal extraction	4/224 (1.8)	4/222 (1.8)

^a^ Safety assessment was conducted in all participants with delivery and neonatal outcomes.

## Discussion

This trial demonstrated that exercise following the PEFLOW program during pregnancy decreased the postpartum incidence of SUI in the short term and midterm, which is consistent with previous research. Mørkved et al^23^ reported that the SUI incidence of the training group at 3 months post partum was 32%. Sangsawang and Sangsawang^11^ reported that the SUI incidence of the exercise group at 37 weeks’ gestation was 27.3%. The incidence of SUI in the exercise group in the present trial was lower than the rate reported in these 2 studies. There are 2 possible reasons for this discrepancy. First, PEFLOW is a training method based on overall posture, enabling women to develop better core strength and a stable posture to cope with the imbalance caused by childbirth. This method also helps the pelvic floor structures maintain good elasticity and resilience, thereby producing therapeutic effects. Second, physical therapists evaluated the patients and adjusted exercise intensity levels every 2 weeks to achieve better outcomes.

It was anticipated that the SUI incidence at 6 weeks post partum could be the lowest among all follow-up points, but in reality, it is not as low as those at the 3-month postpartum follow-up based on conservative imputation. The most reasonable explanation comes from publications indicating that the status of the PFM might not return to normal at 6 weeks post partum due to hormones related to delivery.^24,25,26^ Further analysis revealed that the SUI incidence in the exercise group rebounded at 6-month and 12-month postpartum follow-ups, and the participants with an exercise intensity of 80% or greater achieved the lowest SUI incidence at 3 and 6 months post partum. Taking into consideration that PEFLOW was halted after delivery and referring to publications^27^ indicating that postpartum PFM exercise is beneficial for the recovery of pelvic floor function, we argue that postpartum PEFLOW may help control the rebound in long-term SUI incidence.

Analysis of the MOS score in the exercise and control groups demonstrates an immediate effectiveness of the PEFLOW program in strengthening PFMs, with the evidence showing that the proportion of participants with a MOS score of 4 or higher was higher in the exercise group than the control group at 6 weeks after delivery (17.8% vs 7.9%). Based on previous studies, we recommend that pregnant women engage in twice-weekly low-intensity aerobic exercise sessions (lasting 15 minutes each) combined with daily 10-minute PFMT sessions during pregnancy.^28,29^ Based on our findings, the intensity of PEFLOW is directly associated with immediate and sustainable improvements in both SUI and PFM strength, and continuing postpartum PEFLOW appears necessary to achieve the anticipated sustainable improvements in PFM status and to reduce the incidence of SUI in the long term. Meanwhile, we found no evidence of any adverse effects of PEFLOW during midpregnancy and later pregnancy on the mother or fetus.

### Limitations

The limitations of this study mainly lie in the unfeasibility of blinding the participants in the control group about the intervention, which biases the data from the control group. In addition, this trial, to our knowledge, was the first to examine the effectiveness of PEFLOW; thus, we did not include postpartum PEFLOW and relevant observations in the protocol. For this reason, further study is required.

## Conclusions

In this multicenter RCT, PEFLOW during pregnancy effectively reduced the incidence of postpartum SUI both immediately and sustainably. This finding suggests that PEFLOW may be an effective option for preventing SUI in pregnancy. Additional studies are warranted.

## Notes

Supplement 1.
Trial Protocol

Supplement 2. **eFigure.** Comparison of SUI Incidence Among Different Exercise Intensities in Exercise Group

Supplement 3.
Data Sharing Statement

## References

[1] Haylen BT, de Ridder D, Freeman RM, . An International Urogynecological Association (IUGA)/International Continence Society (ICS) joint report on the terminology for female pelvic floor dysfunction. Int Urogynecol J. 2010;21(1):5-26. doi:10.1007/s00192-009-0976-9 19937315

[2] Qi X, Shan J, Peng L, Zhang C, Xu F. The effect of a comprehensive care and rehabilitation program on enhancing pelvic floor muscle functions and preventing postpartum stress urinary incontinence. Medicine (Baltimore). 2019;98(35):e16907. doi:10.1097/MD.0000000000016907 31464923 PMC6736454

[3] Siafarikas F, Halle TK, Benth JŠ, . Pelvic floor symptoms from first pregnancy up to 8 years after the first delivery: a longitudinal study. Am J Obstet Gynecol. 2022;227(4):613.e1-613.e15. doi:10.1016/j.ajog.2022.06.020 35724758

[4] Delancey JO, Ashton-Miller JA. Pathophysiology of adult urinary incontinence. Gastroenterology. 2004;126(1 suppl 1):S23-S32. doi:10.1053/j.gastro.2003.10.080 14978635

[5] DeLancey JO. Structural support of the urethra as it relates to stress urinary incontinence: the Hammock hypothesis. Am J Obstet Gynecol. 1994;170(6):1713-1720. doi:10.1016/S0002-9378(94)70346-9 8203431

[6] NICE Guidance—urinary incontinence and pelvic organ prolapse in women: management: NICE (2019) urinary incontinence and pelvic organ prolapse in women: management. BJU Int. 2019;123(5):777-803. doi:10.1111/bju.14763 31008559

[7] Reilly ET, Freeman RM, Waterfield MR, Waterfield AE, Steggles P, Pedlar F. Prevention of postpartum stress incontinence in primigravidae with increased bladder neck mobility: a randomised controlled trial of antenatal pelvic floor exercises. BJOG. 2014;121(suppl 7):58-66. doi:10.1111/1471-0528.13213 25488090

[8] Boyle R, Hay-Smith EJ, Cody JD, Mørkved S. Pelvic floor muscle training for prevention and treatment of urinary and fecal incontinence in antenatal and postnatal women: a short version Cochrane review. Neurourol Urodyn. 2014;33(3):269-276. doi:10.1002/nau.22402 23616292

[9] Davenport MH, Nagpal TS, Mottola MF, . Prenatal exercise (including but not limited to pelvic floor muscle training) and urinary incontinence during and following pregnancy: a systematic review and meta-analysis. Br J Sports Med. 2018;52(21):1397-1404. doi:10.1136/bjsports-2018-099780 30337466

[10] Kissler K, Yount SM, Rendeiro M, Zeidenstein L. Primary prevention of urinary incontinence: a case study of prenatal and intrapartum interventions. J Midwifery Womens Health. 2016;61(4):507-511. doi:10.1111/jmwh.12420 26971402

[11] Sangsawang B, Sangsawang N. Is a 6-week supervised pelvic floor muscle exercise program effective in preventing stress urinary incontinence in late pregnancy in primigravid women? a randomized controlled trial. Eur J Obstet Gynecol Reprod Biol. 2016;197:103-110. doi:10.1016/j.ejogrb.2015.11.039 26720598

[12] Soave I, Scarani S, Mallozzi M, Nobili F, Marci R, Caserta D. Pelvic floor muscle training for prevention and treatment of urinary incontinence during pregnancy and after childbirth and its effect on urinary system and supportive structures assessed by objective measurement techniques. Arch Gynecol Obstet. 2019;299(3):609-623. doi:10.1007/s00404-018-5036-6 30649605

[13] Boyle R, Hay-Smith EJ, Cody JD, Mørkved S. Pelvic floor muscle training for prevention and treatment of urinary and faecal incontinence in antenatal and postnatal women. Cochrane Database Syst Rev. 2012;10:CD007471. doi:10.1002/14651858.CD007471.pub2 23076935

[14] Fozzatti MC, Palma P, Herrmann V, Dambros M. Impacto da reeducação postural global no tratamento da incontinência urinária de esforço feminina. Rev Assoc Med Bras (1992). 2008;54(1):17-22. doi:10.1590/S0104-42302008000100015 18392481

[15] Liu T, Hou X, Xie B, . Pelvic incidence: a study of a spinopelvic parameter in MRI evaluation of pelvic organ prolapse. Eur J Radiol. 2020;132:109286. doi:10.1016/j.ejrad.2020.109286 33007519

[16] Fozzatti C, Herrmann V, Palma T, Riccetto CL, Palma PC. Global postural re-education: an alternative approach for stress urinary incontinence? Eur J Obstet Gynecol Reprod Biol. 2010;152(2):218-224. doi:10.1016/j.ejogrb.2010.06.002 20638774

[17] Araujo CC, Marques AA, Juliato CRT. The adherence of home pelvic floor muscles training using a mobile device application for women with urinary incontinence: a randomized controlled trial. Female Pelvic Med Reconstr Surg. 2020;26(11):697-703. doi:10.1097/SPV.0000000000000670 30624250

[18] Hou Y, Feng S, Tong B, Lu S, Jin Y. Effect of pelvic floor muscle training using mobile health applications for stress urinary incontinence in women: a systematic review. BMC Womens Health. 2022;22(1):400. doi:10.1186/s12905-022-01985-7 36192744 PMC9531466

[19] Gao L, Zhang D, Wang S, . Effect of the app-based video guidance on prenatal pelvic floor muscle training combined with global postural re-education for stress urinary incontinence prevention: a protocol for a multicenter, randomized controlled trial. Int J Environ Res Public Health. 2021;18(24):12929. doi:10.3390/ijerph182412929 34948546 PMC8700899

[20] Messelink B, Benson T, Berghmans B, . Standardization of terminology of pelvic floor muscle function and dysfunction: report from the pelvic floor clinical assessment group of the International Continence Society. Neurourol Urodyn. 2005;24(4):374-380. doi:10.1002/nau.20144 15977259

[21] Guralnick ML, Fritel X, Tarcan T, Espuna-Pons M, Rosier PFWM. ICS educational module: cough stress test in the evaluation of female urinary incontinence: introducing the ICS-Uniform Cough Stress Test. Neurourol Urodyn. 2018;37(5):1849-1855. doi:10.1002/nau.23519 29926966

[22] Huang L, Zhang SW, Wu SL, Ma L, Deng XH. The Chinese version of ICIQ: a useful tool in clinical practice and research on urinary incontinence. Neurourol Urodyn. 2008;27(6):522-524. doi:10.1002/nau.20546 18351586

[23] Mørkved S, Bø K, Schei B, Salvesen KA. Pelvic floor muscle training during pregnancy to prevent urinary incontinence: a single-blind randomized controlled trial. Obstet Gynecol. 2003;101(2):313-319. doi:10.1097/00006250-200302000-00018 12576255

[24] Bø K, Næss K, Stær-Jensen J, Siafarikas F, Ellström Engh M, Hilde G. Recovery of pelvic floor muscle strength and endurance 6 and 12 months postpartum in primiparous women—a prospective cohort study. Int Urogynecol J. 2022;33(12):3455-3464. doi:10.1007/s00192-022-05334-y 36048249 PMC9666345

[25] Lin X, Chen J, Pan H, . Comparative magnetic resonance imaging-based study of pelvic floor morphology and function before pregnancy and after primigravida vaginal delivery. BMC Pregnancy Childbirth. 2025;25(1):62. doi:10.1186/s12884-025-07198-8 39856617 PMC11759434

[26] Jiang J, Li C, Liu HY, Zhu ZY. Relationship between abnormal pelvic floor electromyography and obstetric factors in postpartum women: a cross-sectional study. BMC Womens Health. 2024;24(1):239. doi:10.1186/s12905-024-03045-8 38616274 PMC11017559

[27] Beamish NF, Davenport MH, Ali MU, . Impact of postpartum exercise on pelvic floor disorders and diastasis recti abdominis: a systematic review and meta-analysis. Br J Sports Med. 2025;59(8):562-575. doi:10.1136/bjsports-2024-108619 39694630 PMC12013572

[28] García-Sánchez E, Ávila-Gandía V, López-Román J, Martínez-Rodríguez A, Rubio-Arias JÁ. What pelvic floor muscle training load is optimal in minimizing urine loss in women with stress urinary incontinence? a systematic review and meta-analysis. Int J Environ Res Public Health. 2019;16(22):4358. doi:10.3390/ijerph16224358 31717291 PMC6887794

[29] Sun X, Gao L, Zhu H, . Chinese expert consensus on primary prevention for pelvic floor dysfunction during pregnancy. Gynecol Obstet Clin Med. 2023;3(3). doi:10.1016/j.gocm.2023.08.002

