# Supplementary material for: Pelvic Floor Workout for Preventing Stress Urinary Incontinence in Primiparous Women: A Randomized Clinical Trial
Source: JAMA Netw Open. 2026 Apr 15;9(4):e267132. doi: 10.1001/jamanetworkopen.2026.7132 (PMC13084433; doi:10.1001/jamanetworkopen.2026.7132)
Supplement: Supplement 1. — Trial Protocol [file jamanetwopen-e267132-s001.pdf]

Protocol

1. Study Overview

The study is designed as a two-arm, parallel, RCT designed following the Consoli- dated Standards of Reporting Trials (CONSORT). Participants will be recruited from the obstetrics departments of ten hospitals (the centers) including Peking University People's Hospital, Peking University International Hospital, Peking University Shenzhen Hospital, Fangshan Maternal and Child Health Hospital of Beijing, Fengtai Maternal and Child Health Hospital of Beijing, Mentougou District Hospital of Beijing, Zhengzhou Central Hospital Affiliated to Zhengzhou University, Luohe Central Hospital of Henan Province and The First Obstetrics Hospital of Shanghai.

2. Inclusion Criteria

Pregnant women will be eligible for recruitment if they are: (1) 20 to 40 years of ages, (2) pregnant with a single fetus of less than 16 weeks, (3) capable of understanding the research procedures, and (4) able to provide informed consent form in writing.

3. Exclusion Criteria

Participants would be excluded if they had (1) severe complications that the intervention may potentially exacerbate, (2) a history of stress urinary incontinence (SUI) or pelvic organ prolapse (POP), and/or (3) a history of cervical insufficiency, recurrent miscarriage, or induced labor.

4. Randomization and Allocation Concealment

An independent methodological statistician, not involved in recruitment, follow-up, or data analysis, generated a simple randomization sequence using a computer random number generator. A single common sequence was used for all centers. To ensure allocation concealment, this sequence was implemented using sequentially numbered, opaque, sealed envelopes. A dedicated research staff member, separate from the recruiters, safeguarded all envelopes. After the baseline data were collected at enrollment, the participants were randomly assigned to either the Exercise Group (EG) or the Control Group (CG) at a 1:1 ratio via sealed envelopes. Since it was unfeasible to blind the participants and physiotherapists involved in this trial, we trained all investigators on the study components, implementation requirements, detailed procedures, and precautions to minimize study biases. Investigators who conducted patient inspections, examinations, evaluations, and data analysis were blinded to the grouping information.

5. Interventions

Baseline information was collected from participants in both groups at enrollment. Key items included age, pre-pregnancy body mass index (BMI), educational status, occupational categorization, dominant working postures, history of constipation, dominant defecation postures, family history of SUI, and the modified oxford scale (MOS).

Interventions for participants in the EG began with physiotherapists guiding them to perform PEFLOW. Participants in the EG engaged in PEFLOW from the 28th gestational week (gw) until delivery, following a specially designed video guiding app available for download to mobile devices. The app provided health education on pelvic floor protection and a procedural video to guide the PEFLOW exercises. Included in the video were 2 programs for pelvic floor muscle training (PFMT) and global postural exercise. PEFLOW consists of daily

PFMT and two sessions of global postural exercises per week.
PFMT emphasizes the voluntary contraction and relaxation of the PFM surrounding the urethra, vagina, and rectum. It instructs exercisers to synchronize the contraction and relaxation of the PFM with their breathing, specifically through exhalation and inhalation, while avoiding engagement of the abdominal muscles. In the initial PFMT section, participants in EG were required to repeat five times of the primary PFMT procedure: contracting the PFM with moderate strength and holding for 6 to 8 seconds, followed by 6 to 8 seconds of relaxation. After completing this, the participants performed eight repetitions of the second section, consisting of five maximum PFM contractions and relaxations as quickly as possible within 1 second, followed by 10 seconds of relaxation. The first and second PFMT sections together comprised one complete PFMT set.
Global postural exercise in this study consisted of four groups of postures with intensities progressively increasing from level 1 to level 4, specifically designed to match the specific gestational week. Each group includes 4 postures at the same intensity level lasting approximately 30 minutes. Deep breathing at a personal rhythm while maintaining the posture, and holding the maximal contraction of the pelvic floor muscles during exhalation is essential. Participants in EG were encouraged to record the duration for each posture.
Participants in EG were required to perform the overall PEFLOW program in 4 episodes: 28-30, 31-33, 34-36, and 37-40 gw. In each episode, they were instructed to begin the PEFLOW session in postures at level 2 intensity. The intensities of the postures were adjusted upward or downward based on rating of perceived exertion of ≤ 15 or >15, which respectively indicated whether the participant could or could not activate the required muscle contraction while maintaining the current posture.

6. Data Collection and Management

Data regarding the clinical examination during the follow-ups will be collected from all participants in both groups. A questionnaire of demographic characteristics including the age, body mass index (BMI), educational status, working type, working posture, toilet type, PFMT history, constipation history, smoking history, number of gestations, number of abortions, family history of SUI, and family history of POP will be collected from the participants with gestation $\leq$ 16 GWs. The Prolapse/Urinary Incontinence Sexual Questionnaire short form (PISQ-12) will be filled by all participants. Data regarding the MOS, and genital hiatus (gh) and perineal body (pb) measured by pelvic organ prolapse quantitation (POP-Q) of all the participants will be provided by the urogynecologists. Trans- perineal ultrasound using the proprietary software 4D View v 10 (GE Kretz Medizintechnik) will be conducted for all the participants to examine the residual urine, the thickness of pb, detrusor muscle, and levator ani muscle (LAM), and the diameters of the levator hiatus and levator hiatus area. Urinary leakage symptoms, and frequency of PFMT and GPR performance, will be taken from all the enrolled participants respectively at the 28th and 37th GW and at the delivery. Those who have urinary leakage symptoms will fill out the Incontinence Quality of Life (I-QOL) and the International Consultation on Incontinence Questionnaire-urinary incontinence short form (ICIQ-UI SF), and Overactive Bladder Symptom Scores (OABSS) will be assigned to participants who have symptoms of urgency urinary incontinence (UUI). Evaluation of the PFM strength via MOS, stress test, and gh and pb measured by POP-Q will be conducted on all participant in both groups on the 37th GW. The delivery type, the duration of the second

labor stage, the perineal laceration classification, the visual analog scale (VAS) score, neonatal Apgar score and the neonatal weight will be recorded during delivery. The visual analog scale (VAS) will be scored at 24 h and 48 h after the delivery to measure the pain degree by scoring 0 to 10. Fetal/neonatal safety will be evaluated with indicators such as the fetal movement, obstetric ultrasound, fetal heart rate, electronic fetal monitoring, neonatal Apgar score, and obstetric examination.
Four follow-ups will be conducted on all participants respectively on the 6th week and in the 3rd, 6th and 12th month postpartum. To conduct the multiple physical valuations in a time-saving way, it is proposed to check the participant in the following sequence: first, check the stress test, POP-Q, MOS when the bladder is half-full; second, conduct the pelvic floor ultrasound examination and the pelvic floor electrophysiological test (Electron-IC Concept Lignon Innovation Co., Montpellier, France) with the emptied bladder; and last, fill the PISQ-12. The pelvic floor electrophysiological indicators mainly include the vaginal resting pressure (cmH2O), the vaginal maximum contraction pressure (cmH2O) and PFM fatigue (%). I-QOL, ICIQ-UI SF, and OABSS will be provided to participants who show symptoms of urinary leakage or UUI as appropriate (Table 1).

Table 1. Baseline screening, assessment, and follow-up schedule.

	At Pregnancy				At Postpartum			
	**<16th Week**	**28th Week**	**37th Week**	**Delivery**	**6th Week**	**3rd Month**	**6th Month**	**12th Month**
Follow-up schedule (week)	0	±1	±1	±1	±1	±2	±2	±2
Informed consent	•							
Demographic data collection	•							
Urinary leakage symptoms	•	•	•	•	•	•	•	•
Training frequency	•	•	•	•	•	•	•	•
Pb and gh	•		•					
Stress test	•		•		•	•	•	•
POP-Q staging					•	•	•	•
The modified Oxford Scale	•		•		•	•	•	•
The duration of the second labor stage				•				
The perineal laceration classification				•				
VAS				•				
Fetal/natal conditions	•	•	•	•				
Pelvic floor ultrasound	•				•	•	•	•
Pelvic floor electrophysiological test					•	•	•	•
PISQ-12	•				•	•	•	•
ICIQ-UI SF		▲	▲		▲	▲	▲	▲
OABSS		▲	▲		▲	▲	▲	▲
I-QOL		▲	▲		▲	▲	▲	▲

•: Indicates mandatory items; ▲: Investigator will decide whether to perform the test according to clinical signs or clinical evaluation. Pb: perineal body; gh: genital hiatus; POP-Q: pelvic organ prolapse quantitation; VAS: visual analog scale; PISQ-12: Prolapse/Urinary Incontinence Sexual Questionnaire short form; ICIQ-UI SF: the International Consultation on Incontinence Questionnaire-urinary incontinence short form; OABSS: Overactive Bladder Symptom Scores; I-QOL: the Incontinence Quality of Life.

Gynecologic examination, pelvic floor ultrasound, pelvic electromyography, and fetal safety will be evaluated respectively by the urogynecologists, ultrasound professors, electromyography nurses, and obstetric professors in each hospital, who will be trained in the Peking University People’s Hospital before the launch of the study. A program that is preset into the video will record times for the full-playing of the video and automatically transfer the

data into the database as the training frequency for each participant. The site assistants will confirm the training frequency of each participant in EG every week by checking the records from the online-guided software, exercise diary, and the video- training. Accomplishment of PFMT will be identified for each participant should she accomplish more than 80% of the training programs prescribed for every week.

Data input devices will be provided to the hospitals to be involved in the research. Paper files will be prepared and kept for each participant to record all data to be collected in the full study stage. Exercise diaries from participants in EG will be used to evaluate their PFMT frequency and training intensity. The database will be protected from access by a password, and the final dataset will only be accessible to the PI, and, under authorization of the PI, to the study coordinator. Desensitized data are available for research purpose only per the permission of the PI.

7. The Outcomes

The primary outcome of this study is the incidence of SUI at six weeks postpartum. The secondary outcomes are: (1) the incidence of SUI at 37 gw, 3 months, 6 months, and 12 months postpartum and (2) pelvic floor muscle (PFM) strength at 37gw, 6weeks, 3months, 6 months, and 12 months postpartum.

SUI was diagnosed if (1) the stress test was positive for involuntary urinary leakage during Valsalva or coughing in the lithotomy position; and/or (2) the response to the question "When does urine leak?" was urinary leakage during coughing, sneezing, and/or physical exercise according to International Consultation on Incontinence Questionnaire-Urinary Incontinence Short Form (ICIQ-UISF). PFM strength during contraction was measured by digital vaginal examination using the five-point MOS: 0, no contraction; 1, flicker; 2, weak; 3, moderate; 4, good; and 5, strong.

8． Sample Size

The sample size was calculated using PASS 2019 software. Qi et al. reported 25.7% of the 8-week postpartum SUI occurrence. Because there is lack of SUI occurrence in 6-week postpartum to refer to, we hypothesize the 6-week postpartum SUI occurrence is about 25.7% and the occurrence of SUI in EG will dropped to 15% after the trial. Based on 10.7% of occurrence difference between two groups at 6-weeks postpartum, a total of 586 participants (293 per group) is required to provide 90% statistic power at the two-sided significance level of 0.05. In considering 20% of the possible dropout, we decided to recruit 734 participants (367 subjects in each group) for the trial.

9. Statistical Analysis

When conducting comparisons, categorical variables were represented by frequencies and percentages. Continuous variables were summarized using means (standard deviations [SD]) or medians (interquartile ranges [IQR]). The Kolmogorov-Smirnov test was conducted to assess the normality of continuous variables. The primary and secondary outcomes were analyzed using "intention-to-treat" (ITT) methods. ITT analyses of the primary outcome were conducted in three dimensions: multiple imputation analysis was performed using logistic regression with five imputations, using the complete variables as auxiliary variables, and combining the multiply imputed datasets using Rubin's formula; conservative imputation analysis was conducted with the "worst" outcome assigned to cases with the relevant missing data; and complete data analysis was performed without imputation. The analyses of

secondary outcomes were conducted based on conservative imputation. The difference in SUI incidence and proportion of MOS**≥4** between the EG and CG at different time points was analyzed using generalized estimating equations. The analyses of SUI incidence among different exercise intensities in EG group was performed using generalized estimating equations, which based on complete data cases. The 95% confidence interval (CI) for the difference between the two groups was calculated using the Wilson procedure with a continuity correction. The Chi-square test or Fisher's exact test was applied to compare the differences in proportions between the two groups. Data analysis for this study was conducted using SPSS 25.0 and GraphPad Prism 9.5.0. A two-sided p-value of less than 0.05 was considered statistically significant.
