# Supplementary material for: Pelvic Floor Workout for Preventing Stress Urinary Incontinence in Primiparous Women: A Randomized Clinical Trial
Source: JAMA Netw Open. 2026 Apr 15;9(4):e267132. doi: 10.1001/jamanetworkopen.2026.7132 (PMC13084433; doi:10.1001/jamanetworkopen.2026.7132)
Supplement: Supplement 2. — eFigure. Comparison of SUI Incidence Among Different Exercise Intensities in Exercise Group [file jamanetwopen-e267132-s002.pdf]

Supplemental Online Content

Gao L, Zhu H, Sun X, et al. Pelvic floor workout for preventing stress urinary incontinence in primiparous women: a randomized clinical trial. *JAMA Netw Open*. 2026;9(4):e267132. doi:10.1001/jamanetworkopen.2026.7132

eFigure. Comparison of SUI Incidence Among Different Exercise Intensities in Exercise Group

This supplemental material has been provided by the authors to give readers additional information about their work.

© 2026 Gao L et al. *JAMA Network Open*

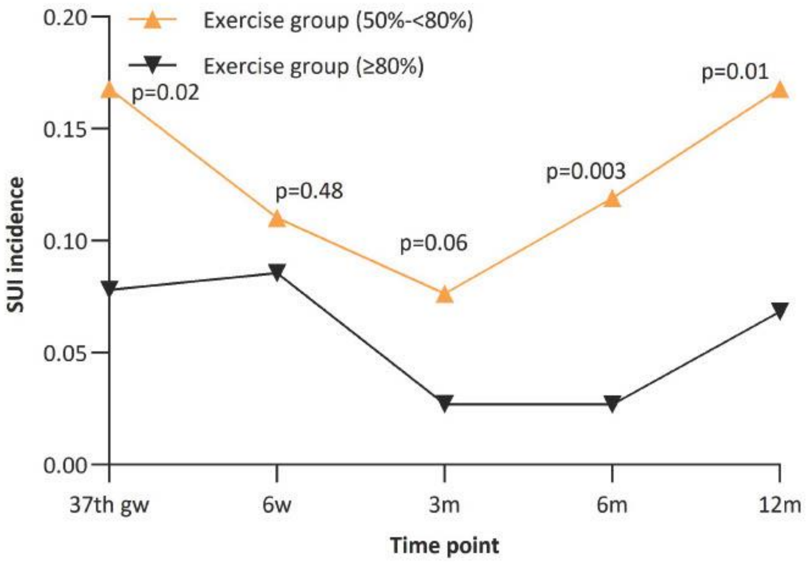

eFigure. Comparison of SUI Incidence Among Different Exercise Intensities in Exercise Group

37th gw, 37th gestational week; 6w, 6 week post partum; 3m, 3 month post partum; 6m, 6 month post partum; 12m, 12 month post partum; SUI, stress urinary incontinence

The analyses of SUI incidence were performed using generalized estimating equations with repeated measures. Chi-squared test was applied to compare the differences in proportions between the two groups.

© 2026 Gao L et al. *JAMA Network Open*
