# Supplementary material for: Pelvic Floor Workout for Preventing Stress Urinary Incontinence in Primiparous Women: A Randomized Clinical Trial
Source: JAMA Netw Open. 2026 Apr 15;9(4):e267132. doi: 10.1001/jamanetworkopen.2026.7132 (PMC13084433; doi:10.1001/jamanetworkopen.2026.7132)
Supplement: Supplement 3. — Data Sharing Statement [file jamanetwopen-e267132-s003.pdf]

Data Sharing Statement

Gao. Pelvic Floor Workout for Preventing Stress Urinary Incontinence in Primiparous Women. *JAMA Netw Open*. Published April 15, 2026. doi:10.1001/jamanetworkopen.2026.7132

Data
Additional Information: Name：Effect of the online-guide direct and indirect pelvic floor muscle training during pregnancy on SUI prevention during the COVID-19 pandemic: protocol for a multicenter, randomized controlled trial URL：https://www.chictr.org.cn/showproj.html?proj=48903 Registration number: ChiCTR2000029618
Data available: Yes
Data types: Data dictionary
How to access data: The data that support the findings of this study are available on request from the corresponding author.
When available: With publication

Supporting Documents
Document types: None

Additional Information
Who can access the data: Researchers whose proposed use of the data has been approved
Types of analyses: For analysis of the preventive effect of pelvic floor muscle exercises during pregnancy on postpartum stress urinary incontinence
Mechanisms of data availability: After received approval from corresponding author
